# Transcriptomic Analysis of Age-Associated Periventricular Lesions Reveals Dysregulation of the Immune Response

**DOI:** 10.3390/ijms21217924

**Published:** 2020-10-25

**Authors:** Motaz M. Fadul, Paul R. Heath, Johnathan Cooper-Knock, Julian M. Kurz, Hayder A. Al-Azzawi, Zarki Ali, Taylor Smith, Fiona E. Matthews, Carol Brayne, Stephen B. Wharton, Julie E. Simpson

**Affiliations:** 1 Sheffield Institute for Translational Neuroscience, University of Sheffield, Sheffield S10 2HQ, UK; mmfadul1@sheffield.ac.uk (M.M.F.); p.heath@sheffield.ac.uk (P.R.H.); j.cooper-knock@sheffield.ac.uk (J.C.-K.); jmkurz1@sheffield.ac.uk (J.M.K.); haaal-azzawi1@sheffield.ac.uk (H.A.A.-A.); zali7@sheffield.ac.uk (Z.A.); tsmith11@sheffield.ac.uk (T.S.); s.wharton@sheffield.ac.uk (S.B.W.); 2 Institute of Public Health, University of Cambridge, Cambridge CB2 0SR, UK; Fiona.Matthews@newcastle.ac.uk (F.E.M.); cb105@medschl.cam.ac.uk (C.B.); 3 Institute of Health and Society, University of Newcastle, Newcastle upon Tyne NE4 5PL, UK

**Keywords:** periventricular lesions, transcriptomic profiling, nanostring, immune response

## Abstract

White matter lesions (WML) are a common feature of the ageing brain associated with cognitive impairment. The gene expression profiles of periventricular lesions (PVL, *n* = 7) and radiologically-normal-appearing (control) periventricular white matter cases (*n* = 11) obtained from the Cognitive Function and Ageing Study (CFAS) neuropathology cohort were interrogated using microarray analysis and NanoString to identify novel mechanisms potentially underlying their formation. Histological characterisation of control white matter cases identified a subgroup (*n* = 4) which contained high levels of MHC-II immunoreactive microglia, and were classified as “pre-lesional.” Microarray analysis identified 2256 significantly differentially-expressed genes (*p* ≤ 0.05, FC ≥ 1.2) in PVL compared to non-lesional control white matter (1378 upregulated and 878 downregulated); 2649 significantly differentially-expressed genes in “pre-lesional” cases compared to PVL (1390 upregulated and 1259 downregulated); and 2398 significantly differentially-expressed genes in “pre-lesional” versus non-lesional control cases (1527 upregulated and 871 downregulated). Whilst histological evaluation of a single marker (MHC-II) implicates immune-activated microglia in lesion pathology, transcriptomic analysis indicates significant downregulation of a number of activated microglial markers and suggests established PVL are part of a continuous spectrum of white matter injury. The gene expression profile of “pre-lesional” periventricular white matter suggests upregulation of several signalling pathways may be a neuroprotective response to prevent the pathogenesis of PVL.

## 1. Introduction

Age-associated white matter lesions (WML) appear as hyperintensities on T2-weighted magnetic resonance images (MRI) and are frequently observed in people aged over 65 years [1]. These WML are often interpreted as a surrogate for small vessel disease and are associated with an increased risk of dementia [2,3,4]; the impact of WML on cognition is related to their neuroanatomical locations [5].

WML can be subclassified as periventricular (PVL) or deep subcortical lesions (DSCL), which both share similar histopathological features, including myelin attenuation, axonal damage, astrogliosis and microglial activation, as reviewed in [6,7,8]. However, differences in the cellular pathology profiles of these WML have also been reported, particularly in relation to their microglial phenotype. Microglia share functions with tissue macrophages, including surveillance, antigen presentation and phagocytosis [9]. Previous studies suggest microglia within PVL have a predominantly pro-inflammatory immune reactive phenotype, associated with the expression of the major histocompatibility complex class II (MHC-II) and co-stimulatory molecule B7-2 (CD80) [10], while microglia within DSCL have a phagocytic phenotype associated with the abundance of CD68^+^ amoeboid microglia [11,12].

The exact mechanism(s) underlying the pathogenesis of WML remains unknown. However chronic cerebral hypoperfusion [13,14], dysfunction of the blood brain-barrier (BBB) [15,16,17] and cortical pathologies have been linked to their formation [18]. WML show arteriolar thickening, perivascular widening, capillary loss and perivenous collagenosis, with DSCL in particular associated with the expression of high levels of a number of hypoxia response molecules, suggesting that vascular insufficiency and consequent hypoperfusion underlie their formation [13]. This is further supported by transcriptomic profiling of DSCL, which has confirmed increased expression of hypoxia-related genes, and also identified dysregulation of immune-regulatory genes, including those involved with antigen presentation, pro-inflammatory cytokine signalling and phagocytosis [19].

Transcriptomic analyses enable the identification of relevant gene expression changes that are often missed in targeted approaches. While histological studies suggest BBB dysfunction, serum protein accumulation and microglial activation underlie the pathogenesis of PVL [10,15,20], to date no studies have employed transcriptomic analysis to characterise their profile. Therefore, the current study interrogated the gene expression profile of PVL using two independent approaches to identify novel mechanisms potentially underlying their formation.

## 2. Results

### 2.1. Histological Characterisation of Periventricular White Matter

**Figure 1:**
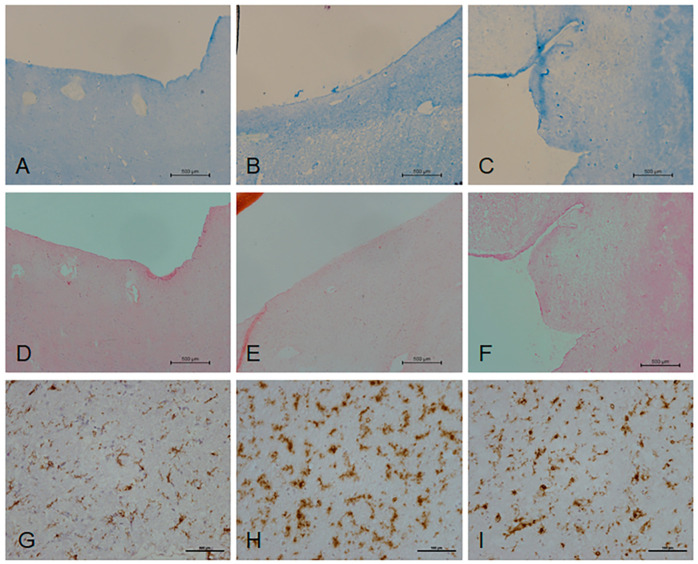
Histological characterisation of periventricular white matter. Luxol fast blue (LFB) (**A**,**B**) and H&E staining (**D**,**E**) of radiologically normal appearing white matter demonstrated a regular pattern of myelin staining across the periventricular region. In contrast, LFB (**C**) and H&E staining (**F**) of radiologically identified lesional cases displayed a band of periventricular demyelination. MHC-II immunostaining of radiologically normal appearing white matter identified cases with minimal MHC-II^+^ microglia ((**G**), control) but also cases with high levels of MHC-II immunoreactivity ((**H**), “pre-lesional”). Radiologically identified periventricular lesions (PVL) contained high levels of MHC-II^+^ microglia (**I**). Scale bar represents 500 µm in (**A**–**F**) and 100 µm in (**G**–**I**).

MRI of the formalin-fixed hemisphere was used to guide sampling of the contralateral frozen periventricular white matter, and histological characterisation of the sampled frozen blocks was performed to confirm the tissue status as non-lesional control periventricular white matter or PVL. These frozen blocks were then sampled for transcriptomic analysis. All non-lesional control cases displayed preservation of myelin throughout the periventricular region and an intact ependymal lining (Figure 1A,B,D,E). In contrast, all PVL cases were characterised by a reduction in luxol fast blue (LFB) staining, indicative of myelin attenuation (Figure 1C), and partial denudation of the ependyma in four out of seven of the cases (Figure 1F).

To further characterise the periventricular white matter samples, activated microglia were visualised using MHC-II, which immunolabelled the microglial cell body and proximal processes. Seven cases of radiologically and histologically-rated non-lesional control white matter contained low levels of MHC-II immunoreactive microglia throughout the periventricular region (Figure 1G); however, four cases contained high levels of MHC-II expression (Figure 1H). While the MRI and histological characterisation had indicated that there was no demyelination, the immunoreactive profile of MHC-II suggested microglial activation; therefore, the non-lesional group was subsequently subdivided into non-lesional control (no demyelination; low levels of MHC-II immunoreactivity, *n* = 7) and “pre-lesional” cases (no demyelination; high levels of MHC-II^+^ microglia, *n* = 4). Seven cases of radiologically and histologically-identified PVL contained high levels of MHC-II^+^ microglia (Figure 1I).

### 2.2. Microarray Analysis

**Figure 2:**
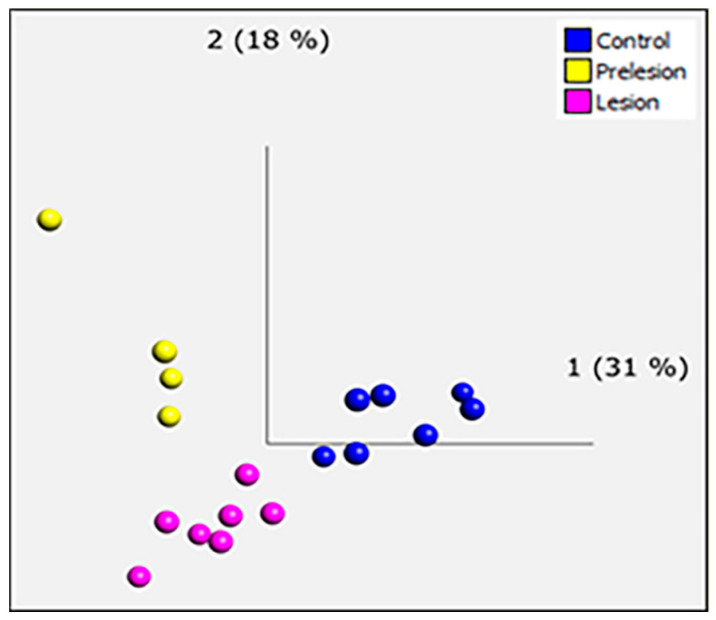
Principle component analysis (PCA) of ageing periventricular transcriptomic datasets. The PCA plot of control (blue), “pre-lesion” (yellow) and PVL (pink) shows a separation of differentially-expressed genes between the groups.

Based on the bioanalyser RNA quality profile, transcriptomic analysis of 18 periventricular white matter cases (seven control, four “pre-lesional” and seven PVL) was performed using Human Genome U133 Plus 2.0 Arrays, which contain at least one probe for each gene and recognize over 39,000 genes. Principal component analysis (PCA) showed clear separation of the three groups and confirmed there were no sample outliers (Figure 2); quality control (QC) measures were within an acceptable range. After setting the stringency parameters to fold change ≥ 1.2 and *p* value ≤ 0.05, 2256 genes were significantly differentially expressed in PVL compared to non-lesional control (1378 upregulated and 878 downregulated); 2649 were significantly differentially-expressed genes in “pre-lesional” cases compared to PVL (1390 upregulated and 1259 downregulated); and 2398 genes were significantly differentially expressed in “pre-lesional” versus non-lesional control cases (1527 upregulated and 871 downregulated). The microarray datasets are freely available at the Gene Expression Omnibus (GEO) public database (accession code GSE157363).

In PVL compared to non-lesional control periventricular white matter, functional grouping analysis with the highest stringency settings identified enrichment of genes that were associated with MHC antigen processing and presentation, major histocompatibility II receptor activity and collagen IV. Kyoto Encyclopedia of Genes and Genomes (KEGG) pathway analysis in the Database for Annotation, Visualization and Integrated Discovery (DAVID) identified 53 significantly dysregulated pathways (Supplementary Materials, Table S1), including immune response-associated pathways (Table 1). In PVL, genes associated with antigen processing and presentation (MHC-II) were significantly downregulated, including *HLA-DRA* (probeset 210982_s_at, FC = −1.71, *p* = 0.0038) and *CD74* (probeset 1567627_at, FC = −2.67, *p* = 0.0068). Significant downregulation of genes associated with the phagosome was also detected, including *FCGR1A* (probeset 216951_at, FC = −2.12, *p* = 0.0164).

In “pre-lesional” compared to non-lesional control periventricular white matter, functional grouping analysis revealed enrichment of genes associated with calcium signalling, glutamate receptor activity, GABA receptor activity and MHC classes I/II-like antigen recognition protein. KEGG pathway analysis identified 79 significantly dysregulated pathways (Supplementary Materials, Table S2), including immune response-associated signalling pathways (including calcium, cAMP and MAPK) and synaptic pathways (including glutamatergic, dopaminergic and cholinergic synapses) (Table 2). In “pre-lesional” cases, multiple genes associated with the phagosome, antigen processing and antigen presentation were downregulated, including *HLA-DPA1* (probeset 213537_at, FC = −2.72, *p* = 0.0004) and *HLA-DQB1* (probeset 211654_x_at, FC = −1.76, *p* = 0.0046). Upregulation of multiple genes associated with calcium signalling and synaptic signalling was found, including calcium voltage-gated channel subunit alpha 1E (*CACNA1EI*) (probeset 236013_at, FC = 4.46, *p* = 0.001; probeset 244256_at, FC = 4.11, *p* = 0.001; probeset 240650_at, FC = 1.31, *p* = 0.016; probeset 208432_s_at, FC = 1.62, *p* = 0.012) and calcium/calmodulin-dependent protein kinase IV (*CAMK4*) (probeset 229029_at, FC = 2.74, *p* = 0.0008; probeset 241871_at, FC = 3.31, *p* = 0.0008), along with upregulation of genes associated with GABAergic synaptic signalling, including gamma-aminobutyric acid (GABA) A receptor alpha 5 *GABRA5* (probeset 206456_at, FC = 7.1, *p* = 0.0002; probeset 215531_s_at, FC = 3.89, *p* = 0.001; probeset 217280_x_at, FC= 1.6, *p* = 0.009).

In “pre-lesional” periventricular white matter compared to PVL, functional grouping analysis identified an enrichment of genes associated with GABA receptor activity, adenylate cyclase activity, metallothionein and neurotransmitter gated ion channels. KEGG pathway analysis identified 38 significantly dysregulated pathways (Supplementary Materials, Table S3), including signalling pathways (including calcium, cAMP, cGMP and MAPK) and synaptic pathways (including glutamatergic, GABAergic and dopaminergic synapses) (Table 3). Significant upregulation of multiple genes encoding the calcium signalling pathway was detected in “pre-lesional” periventricular white matter, including upregulation of calmodulin 1 and calmodulin 2 (*CALM1* and *CALM2*) (probeset 213688_at, FC = 1.59, *p* = 0.016) and calcium/calmodulin-dependent protein kinase II alpha (*CAMK2A*) (probeset 213108_at, FC = 1.67, *p* = 0.043; probeset 207613_s_at, FC = 7.06, *p* = 0.008); and upregulation of genes associated with glutamatergic signalling, including glutamate receptor, ionotropic, N-methyl D-aspartate 1 (*GRIN1*) (probeset 205915_x_at, FC = 1.45, *p* = 0.011; probeset 211125_x_at, FC = 1.65, *p* = 0.041; probeset 210781_x_at, FC = 1.82, *p* = 0.018), glutamate receptor, ionotropic, AMPA 2 (*GRIA2*) (probeset 241172_at, FC = 1.55, *p* = 0.015) and *GRIA3* (probeset 1569290_s_at, FC = 1.6, *p* = 0.017; probeset 217565_at, FC = 1.87, *p* = 0.021; probeset 208032_s_at, FC = 2.38, *p* = 0.0005; probeset 230144_at, FC = 2.66, *p* = 0.004; probeset 206730_at, FC = 2.94, *p* = 0.006).

### 2.3. EnrichR

The top 1000 significantly differentially-expressed genes in the PVL versus control non-lesional white matter dataset were imported into EnrichR to check their relationships with KEGG pathways, biological processes, panther pathways and the human gene atlas. The top pathway or functional group identified in each analysis included: antigen processing and presentation (KEGG pathway *p* = 6.77 × 10^−6^); inflammation (panther pathway, *p* = 0.0001); and MHC-II (cellular component *p* = 0.0001). The top 10 pathways/functional groups identified by EnrichR are shown in Table 4.

### 2.4. Computational Deconvolution of Microglial Gene Expression

**Figure 3:**
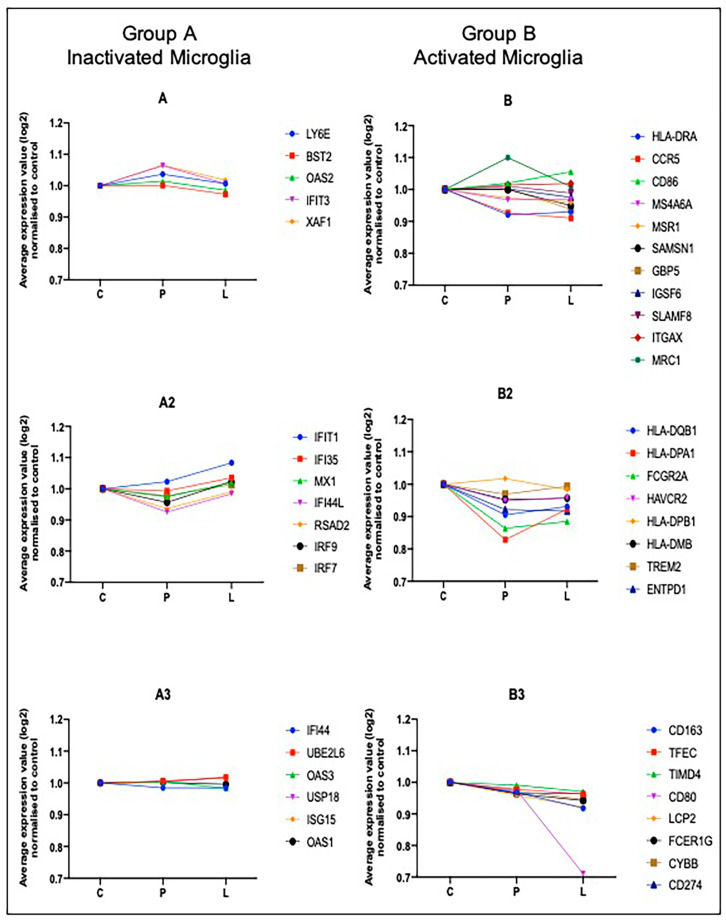
Graphical representation of gene annotations in activated and inactivated microglia in ageing periventricular white matter. *BST2* was used to identify the co-expressed genes associated with inactivated microglia (**A**–**A3**). For activated microglia, *TREM2*, *HLA-DRA*, *ENTPD1*, *CD80*, *CD86*, *CCR5*, *CD274*, *ITGAX*, *TIMD4* and *MRC1* were selected (**B**–**B3**). C: non-lesional control white matter; P: “pre-lesional”; L: periventricular lesion.

To focus the results on changes in the microglial phenotype, bone marrow stromal cell antigen 2 (*BST2),* also referred to as *CD317* or tetherin, was used to identify the co-expressed genes associated with inactivated microglia (group A), while for activated microglia, *TREM2, HLA-DRA, ENTPD1, CD80, CD86, CCR5, CD274, ITGAX, TIMD4* and *MRC1* were selected (group B). In both activated and inactivated microglia groups, 17 genes were co-expressed with the gene set selected (Table 5), which showed similar patterns of expression in the three groups: non-lesional control, “pre-lesion” and PVL (Figure 3). Overall analysis of the co-expressed genes suggested similar levels of activated and inactivated microglia within PVL and “pre-lesions” compared to non-lesional control white matter, with the exception of *CD80* which showed a marked decrease in expression in PVL (Figure 3).

### 2.5. Validation of Candidate Gene Expression by NanoString nCounter

Nanostring is a hybridisation-multiplex digital technology which uses capture probes and unique color-coded barcode reporter probes to measure a broad range of mRNA in a single reaction without the need of reverse transcription of mRNA or amplification of cDNA.

NanoString data analysis was employed to validate a panel of candidate gene expression changes identified in the microarray analysis of PVL versus non-lesional control white matter. NanoString analysis confirmed the significant differential expression of seven genes (*CCL3 p =* 0.008; *CCR5 p =* 0.0002; *CD74 p =* 0.013; *CX3CR1 p =* 0.01; *IL1B p =* 0.0007; *JUN p =* 0.008; *PTGS2 p =* 0.007), and identified 19 genes which showed the same directional changes to the microarray findings, although not reaching statistical significance (Table 6).

### 2.6. Validation of Candidate Gene Expression by Immunohistochemistry

**Figure 4:**
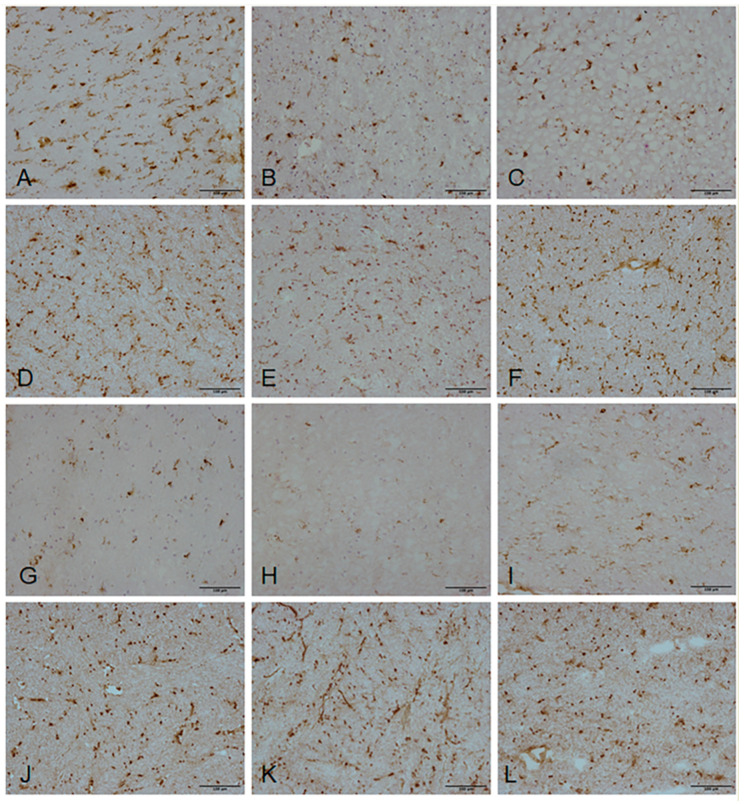
Histological validation candidate gene expression changes. Expression of proteins encoded by candidate genes highlighted by both microarray and NanoString datasets was assessed. (**A**–**C**) CD74, (**D**–**F**) IL-1β, (**G**–**I**) CD163 and (**J**–**L**) CD86 immunolabelled cell bodies and processes of microglia in all periventricular white matter groups: non-lesional control white matter (**A**,**D**,**G**,**J**); “pre-lesional” white matter (**B**,**E**,**H**,**K**); and PVL (**C**,**F**,**I**,**L**). Scale bar represents 100 µm.

**Figure 5:**
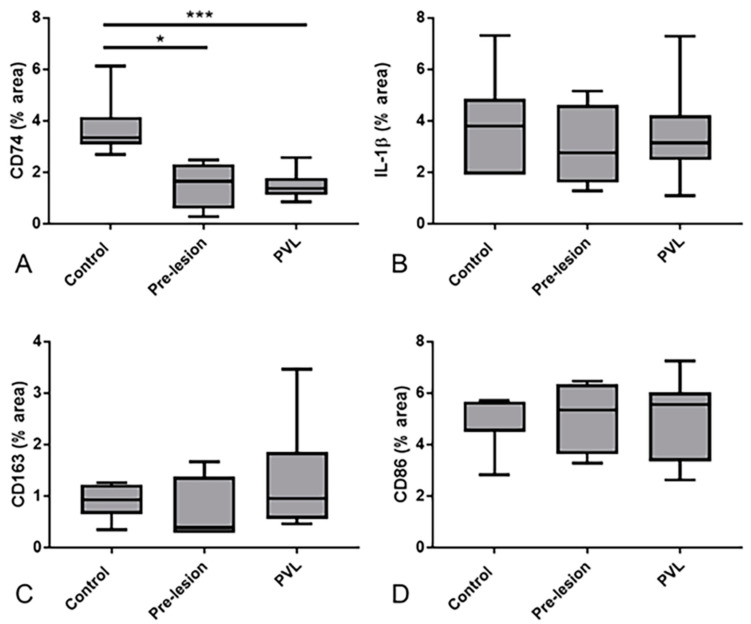
Quantitation of the expression of candidates in periventricular ageing white matter. The percentage area immunoreactivity of the protein encoded by a panel of candidate genes was assessed in the three periventricular white matter groups. (**A**) CD74 expression was significantly higher in non-lesional control periventricular white matter compared to both “pre-lesional” (*p* = 0.033) and PVL (*p* = 0.003). The immunoreactive profiles of (**B**) IL-1β, (**C**) CD163 and (**D**) CD86 did not significantly differ across the three groups. * *p* < 0.05, *** *p* < 0.005.

To further validate our transcriptomic findings, we selected a panel of four biologically relevant genes from pathways highlighted by both the microarray and NanoString datasets (*CD74*, *IL1B*, *CD163*, *CD86*). Expression of proteins encoded by the genes of interest were examined by immunohistochemistry (Figure 4), and the immunoreactive area of CD74, IL-1β, CD163 and CD86 staining in the periventricular white matter was quantified within each group (Table 7). CD74 immunolabelled microglia with a ramified morphology and were mainly present in the non-lesional control group (Figure 4A–C). Expression of CD74 significantly differed across the three groups (*p* = 0.002), with non-lesional control periventricular white matter containing significantly higher levels of CD74 immunoreactivity compared to both “pre-lesional” (*p* = 0.033) and PVL (*p* = 0.003) (Figure 5A). IL-1β, CD163 and CD86 immunoreactivity was predominantly associated with the cell bodies and extending processes of cells morphologically resembling microglia. Expression of IL-1β (*p* = 0.7), CD163 (*p* = 0.38) and CD86 (*p* = 0.63) immunoreactivity did not significantly vary across the three groups (Figure 5B–D).

## 3. Discussion

Age-associated WML correlate with reductions in information processing speed and executive function, and are associated with an increased risk of developing dementia [8,21]. Histological characterisation and transcriptomic profiling of DSCL suggest that vascular insufficiency and hypoperfusion underlie their formation [13], but to date no comparable studies have interrogated the gene expression profile of PVL. The current study employed two independent approaches to characterise the transcriptomic signature of PVL, aiming to identify potential mechanisms underlying their pathogenesis. We demonstrate that established, demyelinated PVL are associated with dysregulation of the immune response, whilst increased signalling is a feature of “pre-lesional” periventricular white matter and may represent attempts to prevent lesion formation.

PVL are characterised by dysfunction of the BBB and significant levels of immune-activated MHC-II^+^ microglia [10,15,17], suggesting that a loss of cerebrovascular integrity and the resulting microglial activation contribute to lesion pathology. In the current study, radiologically identified PVL and non-lesional control periventricular white matter cases underwent histological validation, including assessment of MHC-II immunoreactivity, prior to transcriptomic profiling. As expected, demyelination and MHC-II^+^ microglia are prominent features of PVL; however, a subgroup of radiologically normal periventricular white matter cases were identified, which showed no evidence of demyelination but contained moderately high levels of MHC-II^+^ microglia. The transcriptomic profile of these cases was subsequently assessed independently and termed “pre-lesional,” although it should be acknowledged that it cannot be definitively known whether these cases would have progressed to develop clinically relevant PVL. Extensive detailed histological characterisation studies are required to fully elucidate the profile of these “pre-lesions”.

Microarray analysis indicates that the transcriptome of “pre-lesional” cases is distinct from radiologically and histologically control-grade white matter from non-lesional cases, and similarly to PVL, is associated with the dysregulation of immune function-associated genes. In contrast to increased immunohistochemical detection of MHC-II, several *HLA*-associated transcripts are significantly down-regulated in both “pre-lesion” and PVL compared to non-lesional control white matter. It should, however, be noted that mRNA and protein expression can be decoupled in time [22,23], and that transcriptomic profiling of post-mortem tissue indicates the gene expression profile at one timepoint. While this discordant result appears to argue against the use of transcriptomic profiling technologies to identify potential biologically relevant gene expression changes, it has been shown that differentially-expressed transcripts have significantly higher correlations between mRNA and protein levels when compared to transcripts that are not differentially expressed [23], giving confidence for the use of mRNA profiling in the current study. Indeed CD74—a protein that associates with MHC-II and is an important chaperone that regulates antigen presentation for the immune response—is significantly down-regulated in “pre-lesional” cases and PVL, at both the mRNA and protein levels.

As gene expression profiling was conducted on total tissue extracts, which may have masked cell-specific changes in the transcriptome, we performed a computational deconvolution to interrogate changes in gene expression specific to microglial phenotypes within “pre-lesions” and PVL. BST2 (CD317) is associated with inactivated microglia [24] and diminishes the expression of inflammatory genes [25]; therefore, BST2 was selected to identify the co-expressed genes associated with inactivated microglia. The phenotypic transformation from homeostatic microglia towards reactive microglia in dementia pathologies is associated with TREM2 [26], HLA-DRA [27], ENTPD1 (CD39) [28], CD80 (B7-1) [29], CD86 (B7-2) [10], CCR5 [30], CD274 [31], ITGAX (CD11c) [32], TIMD4 [33] and MRC1 [34]. Therefore, these candidates were used in the deconvolution analysis to identify co-expressed genes associated with active microglia in “pre-lesions” and PVL. Overall, our data are consistent with similar levels of both active and inactive-associated microglial transcripts being present in both “pre-lesions” and PVL, with the exception of *CD80* (expressed by antigen presenting cells), which showed a marked decrease in expression in PVL. Overall, our data demonstrate a heterogenous microglial population within periventricular white matter, highlighting the microglial diversity in the periventricular regions of the ageing brain and the need to examine an extensive panel of markers when assessing the microglial phenotype.

Expression of a panel of candidate gene expression changes identified in the microarray analysis of PVL compared to control white matter was validated using NanoString. This novel, robust profiling technology has the ability to analyse low RNA quality and enables the quantification of mRNA without the use of reverse transcription or amplification of cDNA [35], which is advantageous when assessing post-mortem material. In PVL compared to the non-lesional control group, 65% of the genes assessed displayed a non-significant but similar directional change between both platforms, and 24% of the genes revealed a significant difference with similar directional change, validating the findings of the array analysis. Among the validated gene expression changes, significant down-regulation of the chemokine receptors *CCR5* and *CX3CR1*, the chemokine *CCL3* and pro-inflammatory cytokine *IL1B* are features of established PVL. Expressions of these chemoattractant cytokines and receptors are a prominent feature of active multiple sclerosis lesions and their animal models, where they are primarily associated with active microglia and ongoing demyelination [36,37,38]; in contrast, their levels significantly decrease in established PVL.

Cerebral white matter supports the bidirectional transfer of signals between the cortex and subcortical structures, where dysregulation of signalling impacts communication and cognitive function [21]. Comparison of the transcriptomic profile of “pre-lesional” white matter with both control white matter and PVL indicates significant upregulation of signalling and synaptic pathways. Loss of white matter integrity following ischaemic stroke in aged mice is mainly attributed to Ca^2+^ dyshomeostasis, which ultimately induces oligodendrocyte loss and axonal disruption [39]; therefore, the significant increase in calcium signalling in “pre-lesional” white matter may represent a potential neuroprotective mechanism to prevent lesion formation. Axons release vesicles containing glutamate during the propagation of action potentials [40]. Glutamate receptors are expressed by mature oligodendrocytes [41], and communication between axons and myelin has led to the axon–myelin–synapse hypothesis [42], wherein glutamate signalling is a mechanism whereby adaptive myelination occurs in electrically active axons. The current study demonstrates an increase in synaptic signalling is a feature of “pre-lesions” which may reflect a mechanism to maintain myelin integrity and prevent lesion pathogenesis.

The current study performed transcriptomic profiling of a small number of cases (seven non-lesional control, four “pre-lesional” and seven PVL); however, it should be acknowledged that interrogation of 12 or more cases per group may be required to detect all of the significant differentially-expressed genes [43]. Therefore, as some gene expression changes may not have been detected, future research on a larger cohort of cases is required. A further limitation of the current study is the interpretation of the transcriptomic datasets with a bias towards assessing the microglial phenotype associated with ageing periventricular white matter pathology. The total periventricular region isolated by LCM will have contained a heterogeneous population of cells. While several of the candidate gene expression changes are associated with microglial functions and phenotypes, including *HLA*-associated transcripts, they are not uniquely associated with microglia and may reflect changes in the transcriptomic profiles of other cells, for example, astrocytes. Future research is required to confirm which cell population(s) are associated with the candidate genes identified in study.

Age-associated PVL are significantly associated with cognitive decline in the ageing population, but very little is known about the mechanisms underlying the pathology of these lesions. Overall, our data suggest that established PVL are part of a continuous spectrum of white matter injury, and that increased signalling in “pre-lesional” white matter may be a neuroprotective mechanism to prevent the pathogenesis of PVL. Future studies focussing on these pathways is required, as they may represent a potential therapeutic target to prevent lesion formation.

## 4. Materials and Methods

### 4.1. Human CNS Tissue

Frozen human post-mortem CNS tissue was obtained from the CFAS neuropathology cohort, following multi-centre Research Ethical Committee (REC) approval (REC Reference number 15/SW/0246, approved on 10th August 2015). While this cohort is representative of the ageing population, the current study was conducted with a case-control design, as previously described [7]. MRI of formalin fixed post-mortem coronal brain slices was used to guide case selection and sampling of the contralateral frozen hemisphere, using our previously published protocol [19]. A modified Schelten’s semi-quantitative rating [44], was used to select 7 PVL and 11 radiologically-normal-appearing control periventricular white matter cases (Table 8). All radiologically normal control samples scored 0 in both periventricular and deep subcortical regions. All PVL samples scored 3 in the periventricular MRI rating; three samples scored 0 in the deep subcortical region, three samples scored 3 and one sample scored 5.

### 4.2. Histological Characterisation

Frozen sections (10 μm) were fixed in ice-cold acetone at 4 °C for 10 min and stained with haematoxylin and eosin (H&E) or luxol fast blue (LFB) to confirm the presence of PVL or non-lesional periventricular white matter. Previous studies have shown that PVL contain significantly high levels of MHC-II^+^ microglia [10]; therefore, immunohistochemistry (IHC) was performed to detect this marker of microglial activation using a standard Avidin–biotin complex-horse radish peroxidase (ABC-HRP) approach. Antibody conditions and suppliers are shown in Table 9.

### 4.3. Laser Capture Microdissection (LCM)

Cryosections (10 μm) were fixed in ice-cold acetone for 3 min before staining with Toluidine blue and rinsing with diethylpyrocarbonate (DEPC)-treated water, for 30 s each. Sections were dehydrated in a graded series of ethanol (70%, 95%, 100% for 15 s each), cleared in xylene and left to air dry in an air flow hood for at least 60 min before LCM. The periventricular region of interest was isolated using a PixCell II laser-capture microdissection system (Arcturus BioScience, Mountain View, CA, USA), and CapSure macro caps (Arcturus Engineering, Mountain View, CA, USA). Briefly, LCM of the periventricular white matter was performed using 30 μm laser spot size and a pulse power of 65–90 mW. Following microdissection, RNA was extracted using the PicoPure RNA extraction kit, according to manufacturer’s protocol (Arcturus BioScience, Mountain View, CA, USA). Pre- and post-LCM RNA quality was assessed using a Bioanalyser (Agilent, CA, USA).

### 4.4. Microarray

RNA was prepared according to the GeneChip 3′ IVT express amplification protocol (Affymetrix, Stockport, UK). RNA was reverse transcribed to produce single-stranded cDNA by annealing the total RNA with an oligo-d(T) primer with a T7 polymerase binding site. The cDNA was converted into double-stranded cDNA by DNA polymerase and RNAse H. Biotin-labelled cRNA was made using an in vitro transcription (IVT) labelling kit. Following clean-up and purification of the biotin labelled cRNA, samples were checked using the bioanalyzer to ensure sufficient RNA had been generated (Agilent Bioanalyzer 2100). Samples (12 µg) were analysed using GeneChip Human U133 Plus 2.0 arrays (Affymetrix, Stockport, UK).

### 4.5. Transcriptomic Analysis of Microarrays

Quality control (QC) analysis was performed to confirm amplification, hybridisation, expression of housekeeping genes and signal intensity using Affymetrix Expression Console software (Affymetrix^®^, Stickport, UK). Transcriptome Analysis Console (TAC) software version 4.1.1 (Affymetrix^®^, Stockport, UK) was used to analyse and compare the gene expression profile of the 3 groups. Genes were considered significantly differentially expressed if they showed a minimum fold change (FC) ≥ +/-1.2 and *p* ≤ 0.05. Qlucore Omics Explorer (Qlucore, Lund, Sweden) was used to identify the distribution of the samples using principal component analysis (PCA). The significantly differentially-expressed genes were analysed using the Database for Annotation Visualisation and Integrated Discovery (DAVID) annotation website version 6.8, identifying the key gene expression changes in both pathways and functional groups. Annotated genes were filtered using the highest stringency settings to obtain high specificity and minimizing the rate of false-positives. Subsequent analysis focussed on the top 1000 differentially-expressed genes from each comparison group, and they were analysed using EnrichR, an online tool which aids clustering of the genes based on their similar functions. Affymetrix probe IDs were converted into Entrez Gene IDs using Database for Annotation, Visualisation and Integrated Discovery (DAVID) version 6.8 Gene ID conversion tool (david.ncifcrf.gov).

### 4.6. Deconvolution of the Microglial Transcriptome

To interrogate microglial function within the periventricular region, we performed a computational deconvolution of the transcriptomic data [45]. We used cell-type-specific markers for activated and inactivated microglia based on single-cell mass cytometry of the experimental allergic encephalomyelitis mouse model [24], as shown in Table 10. These markers were used to define co-expression networks which were constructed in GeneMANIA (http://www.genemania.org). The parameters used were as follows: maximum resultant genes: 20; maximum resultant attributes: 10; and the co-expression category used for gene interconnection [46]. As a final validation, co-expression networks focussed on microglial subtypes associated with human CNS cells using an independent dataset (http://www.brainrnaseq.org/) [47].

### 4.7. Validation of Gene Expression Changes—NanoString

Validation of key changes in the gene expression profiles identified by the microarray analysis was conducted using NanoString, an independent platform which enables the quantification of mRNA without the use of reverse transcription or amplification of cDNA [35]. The gene expression profiles of 9 samples (4 control and 5 PVL) were analysed using a customised CodeSet human gene panel consisting of 29 inflammation, antigen processing and presentation related genes, and 3 housekeeping genes (NanoString Technologies, Seattle, WA, USA), as shown in Table 11. Briefly, 50 ng of RNA extracted from each LCM-ed periventricular white matter region was added to the CodeSet in hybridisation buffer and incubated at 65 °C for 22 h in a thermocycler. Raw data (RCC files) were analysed using NanoString data analysis software nSolver v4.0.70 (NanoString Technologies, Seattle, WA, USA). Data were normalized to the geometric mean of housekeeping genes in the designed CodeSet. Both the Affymetrix and NanoString datasets were compared to assess expression of the validation candidates.

### 4.8. Validation of Gene Expression Changes—Immunohistochemistry

To further validate the gene expression changes identified in the microarray analysis, expression of the protein encoded by a panel of candidate genes was assessed by immunohistochemistry using a standard ABC-HRP approach. The antibody details are shown in Table 9. Selected regions of interest were marked on H&E sections and mapped onto consecutive immunostained slides. Quantification of specific immunoreactivity was performed by capturing bright-field microscopic images within the marked area using a Nikon Eclipse 80i microscope. The image was thresholded and the immunoreactive area of the field determined per total area examined. Image capture and analysis was performed using Analysis^D software (Nikon, Kingston Upon Thames, UK). Immunohistochemical data were not normally distributed and did not show equality of variances between groups using the Shapiro–Wilk test; therefore, analyses were performed using non-parametric methods. Statistical comparisons of quantitative data between groups were carried out using the Kruskal–Wallis test using SPSS software version 26.0 (SPSS Inc., Chicago, IL, USA). In the case of a significant result, the *p* value was adjusted for multiple testing using the Bonferroni correction method and considered significant if *p* < 0.05.

## Figures and Tables

**Table 1:** KEGG pathway analysis of PVL versus control white matter transcriptomic datasets.

Pathway	Count	*p*-Value
hsa04612: Antigen processing and presentation	20	9.17 × 10^−6^
hsa04145: Phagosome	24	0.003
hsa04672: Intestinal immune network for IgA production	11	0.004
hsa04662: B cell receptor signalling pathway	12	0.026

**Table 2:** KEGG pathway analysis of “pre-lesional” versus control white matter transcriptomic datasets.

Pathway	Count	*p*-Value
hsa04020:Calcium signalling pathway	42	3.38 × 10^−9^
hsa04728:Dopaminergic synapse	34	5.29 ×10^−9^
hsa04724:Glutamatergic synapse	30	6.28 × 10^−8^
hsa04725:Cholinergic synapse	29	1.29 × 10^−7^
hsa04727:GABAergic synapse	23	1.82 × 10^−6^
hsa04612:Antigen processing and presentation	20	1.58 × 10^−5^
hsa04024:cAMP signalling pathway	34	1.54 × 10^−4^
hsa04010:MAPK signalling pathway	40	2.23 × 10^−4^
hsa04145:Phagosome	25	0.002
hsa04062:Chemokine signalling pathway	28	0.005
hsa04151:PI3K-Akt signalling pathway	45	0.005
hsa04660:T cell receptor signalling pathway	17	0.011
hsa04662:B cell receptor signalling pathway	13	0.014
hsa04068:FoxO signalling pathway	19	0.038

**Table 3:** KEGG pathway analysis of “pre-lesional” versus PVL transcriptomic datasets.

Pathway	Count	*p*-Value
hsa04020:Calcium signalling pathway	46	4.71 × 10^−11^
hsa04724:Glutamatergic synapse	29	3.97 ×10^−7^
hsa04727:GABAergic synapse	21	3.58 × 10^−5^
hsa04725:Cholinergic synapse	23	2.30 × 10^−4^
hsa04310:Wnt signalling pathway	26	3.83 × 10^−4^
hsa04024:cAMP signalling pathway	33	5.36 × 10^−4^
hsa04022:cGMP-PKG signalling pathway	28	5.96 × 10^−4^
hsa04728:Dopaminergic synapse	22	0.0039
hsa04010:MAPK signalling pathway	34	0.0143

**Table 4:** Enrich R analysis of PVL versus control non-lesional white matter transcriptomic datasets. The top 10 pathways/functional groups identified by the analysis are shown below.

Pathway/Functional Group	*p*-Value
**KEGG pathway**	
hsa04612: Antigen processing and presentation	6.77 × 10^−6^
hsa05169: Epstein-Barr virus infection	1.06 × 10^−4^
hsa05215: Prostate cancer	1.49 × 10^−4^
hsa05140: Leishmaniasis	4.58 × 10^−4^
hsa05168: Herpes simplex infection	8.04 × 10^−4^
hsa05332: Graft-versus-host disease	8.61 × 10^−4^
hsa04940: Type I diabetes mellitus	0.0012
hsa04915: Estrogen signalling pathway	0.0015
hsa05330: Allograft rejection	0.003
hsa05164: Influenza A	0.0031
**Panther pathway**	
P00031: Inflammation mediated by chemokine and cytokine signalling	1.20 × 10^−4^
P00053: T cell activation	0.0068
P02756: N-acetylglucosamine metabolism	0.0199
P02776: Serine glycine biosynthesis	0.0199
P00010: B cell activation	0.0217
P00007: Axon guidance mediated by semaphorins	0.0257
P00049: Parkinson disease	0.0359
P00054: Toll receptor signalling pathway	0.0391
P00006: Apoptosis signalling pathway	0.0426
P04386: Histamine H2 receptor mediated signalling pathway	0.0443
**Gene Ontology (cellular component)**	
GO:0042613: MHC class II protein complex	1.24 × 10^−4^
GO:0016607: nuclear speck	3.02 × 10^−4^
GO:0045121: membrane raft	4.73 × 10^−4^
GO:0012507: ER to Golgi transport vesicle membrane	5.67 × 10^−4^
GO:0030671: clathrin-coated phagocytic vesicle membrane	9.54 × 10^−4^
GO:0016606: LYSP100-associated nuclear domain	0.0022
GO:0010445: nuclear dicing body	0.0022
GO:0071601: sphere organelle	0.0022
GO:0016604: nuclear body	0.0022
GO:0035363: histone locus body	0.0023

**Table 5:** Microglial deconvolution. *BST2 (CD317)* was used to identify the co-expressed genes associated with inactivated microglia (group A), while for activated microglia *TREM2*, *HLA-DRA*, *ENTPD1*, *CD80*, *CD86*, *CCR5*, *CD274*, *ITGAX*, *TIMD4* and *MRC1* were selected (group B).

Group A (Inactivated Microglia)	Group B (Activated Microglia)
*ISG15*	*HLA-DPB1*
*MX1*	*HLA-DQB1*
*IFI44L*	*HLA-DMB*
*OAS2*	*HLA-DPA1*
*IFI35*	*LCP2*
*OAS1*	*FCER1G*
*LY6E*	*GBP5*
*IFIT1*	*CYBB*
*IFI44*	*SAMSN1*
*UBE2L6*	*SLAMF8*
*IFIT3*	*MS4A6A*
*IRF7*	*IGSF6*
*XAF1*	*HAVCR2*
*RSAD2*	*TFEC*
*OAS3*	*CD163*
*IRF9*	*FCGR2A*
*USP18*	*MSR1*

**Table 6:** Validation of candidate gene expression by NanoString nCounter.

	Microarray		NanoString	
*Gene*	Fold Change	*p*-Value	Fold Change	*p*-Value
*AKT3*	1.33	0.002 **	1.52	0.127
*CCL2*	−1.7	0.05 *	−1.88	0.290
*CCL3*	−1.31	0.05 *	−16.11	0.008 **
*CCL4*	−1.16	0.060	−5.14	0.098
*CCR5*	−1.49	0.01 *	−2.17	0.0003 ***
*CD163*	−3.27	0.315	−1.07	0.928
*CD74*	−2.67	0.006 **	−1.54	0.013 *
*CD8A*	−1.28	0.016 *	−1.23	0.567
*COL6A3*	−1.62	0.01 *	−1.72	0.074
*CX3CR1*	−1.5	0.005 **	−1.64	0.01 *
*CYBB*	−1.62	0.013 *	−1.17	0.577
*EPSTI1*	−1.31	0.839	−1.39	0.101
*FCER1G*	−1.52	0.076	−1.28	0.265
*FGL2*	−1.77	0.012 *	−1.29	0.190
*FPR1*	−2.39	0.017 *	−2.82	0.066
*HLA-DRA*	−1.71	0.004 **	−1.51	0.071
*IFNAR1*	1.79	0.004 **	1.25	0.190
*IGSF6*	−1.27	0.257	−1.12	0.585
*IKBKB*	1.38	0.839	1.88	0.097
*IL1A*	−1.06	0.206	−1.71	0.061
*IL1B*	−1.34	0.01 *	−4.72	0.007 **
*IRF7*	1.04	0.673	−1.04	0.846
*JUN*	1.33	0.008 **	1.98	0.008 **
*MSR1*	−1.95	0.023 *	−1.06	0.877
*OAS1*	−1.12	0.83	1.14	0.644
*PHF21A*	1.36	0.008 **	1.5	0.121
*PTGS2*	−1.3	0.011 *	−2.59	0.007 **
*RSAD2*	−1.2	0.065	1.09	0.757
*SLAMF8*	−1.03	0.679	−1.48	0.327

* *p* ≤ 0.05, ** *p* ≤ 0.01, *** *p* ≤ 0.001, down-regulated transcripts (pink), up-regulated transcripts (green).

**Table 7:** Expression of CD74, IL-1β, CD163 and CD86 in ageing periventricular white matter. The percentage area of immunoreactivity of each of the markers is shown, median (IQR).

WM Group	Control	Pre-Lesional	PVL
CD74	3.47 (3.08–4.15)	1.66 (0.9–2.14)	1.3 (1.13–1.66)
IL-1β	3.87 (1.91–4.86)	2.76 (1.91–4.07)	3.43 (2.48–4.22)
CD163	0.93 (0.75–1.21)	0.39 (0.29–1.08)	0.95 (0.59–1.32)
CD86	4.55 (4.52–5.66)	5.34 (3.98–6.23)	5.04 (3.35–6.03)

IQR: inter-quartile range.

**Table 8:** Cohort demographics.

Experimental Approach	Classification	Median Age (y) (Range)	Gender (M/F)	PMD (h) (Range)
Microarray	Control (*n* = 11)	84 (70–89)	4/7	11 (6–42)
	PVL (*n* = 7)	89 (85–95)	3/4	28 (7–72)
NanoString	Control (*n* = 7)	84 (70–89)	2/5	14 (6–42)
	PVL (*n* = 5)	88 (85–95)	2/3	28 (18–72)

**Table 9:** Antibody sources and conditions.

Antibody	Isotype	Dilution (Time, Temperature)	Supplier
CD163	Monoclonal Mouse IgG	1:100 (1 h RT)	Bio-Rad, UK
CD68 (PG-M1)	Mouse, IgG_3κ_	1:100 (1 h RT)	Dako, UK
CD74	Polyclonal Rabbit IgG	1:200 (1 h RT)	Sigma, UK
CD80/B7-1	Monoclonal Mouse IgG_1_	1:20 (overnight 4 °C)	R&D Systems, UK
CD86/B7-2	Polyclonal goat IgG	1:20 (overnight 4 °C)	R&D Systems, UK
CX3CR1	Monoclonal Mouse IgG_1_	1:25 (overnight 4 °C)	Biolegend, UK
IL-1β	Polyclonal Rabbit IgG	1:200 (1 h RT)	Proteintech, UK
MHC-II (HLA-DR)	Mouse Monoclonal IgG	1:20 (1 h RT)	Dako, UK

Key: h: hour; RT: room temperature; IL-1β: interleukin 1 beta; κ: Kappa.

**Table 10:** Microglial gene signature: Group A (inactivated) and Group B (activated).

	Gene Symbol	Gene Name
**Group A**	*CD317 (BST2)*	Bone marrow stromal cell antigen 2
**Group B**	*CD39 (ENTPD1)*	Ectonucleoside Triphosphate Diphosphohydrolase 1
	*HLA-DRA*	Major Histocompatibility Complex, Class II, DR Alpha
	*CD86*	Cluster of differentiation 86
	*CD80*	Cluster of differentiation 80
	*CD274*	Cluster of differentiation 274
	*TIMD4*	T Cell Immunoglobulin and Mucin Domain Containing 4
	*CD11C (ITGAX)*	Integrin Subunit Alpha X
	*TREM2*	Triggering Receptor Expressed on Myeloid Cells 2
	*CCR5*	C-C chemokine receptor type 5
	*MRC1*	Mannose Receptor C-Type 1

**Table 11:** Nanostring validation candidates.

Function	Gene Symbol	Gene Name
Inflammation	*AKT3*	V-akt murine thymoma viral oncogene homolog 3/RAC-gamma serine/threonine-protein kinase
	*CCL2*	C-C motif chemokine ligand 2
	*CCL3*	C-C motif chemokine ligand 3
	*CCL4*	C-C motif chemokine ligand 4
	*CCR5*	C-C motif chemokine receptor 5 (gene/pseudogene)
	*COL6A3*	Collagen type VI alpha 3 chain
	*CX3CR1*	C-X3-C motif chemokine receptor 1
	*IFNAR1*	Interferon alpha and beta receptor subunit 1
	*IKBKB*	Inhibitor of nuclear factor kappa B kinase subunit beta
	*IL1A*	Interleukin 1 alpha
	*IL1B*	Interleukin 1 beta
	*JUN*	Jun proto-oncogene, AP-1 transcription factor subunit
	*PTGS2*	Prostaglandin-endoperoxide synthase 2
Antigen processing and presentation	*CD74*	CD74 molecule, HLA class II histocompatibility antigen gamma chain
	*HLA-DRA*	Major histocompatibility complex, class II, DR alpha
	*CD8A*	CD8a molecule, T-cell surface glycoprotein CD8 alpha chain
	*CD163*	CD163 molecule, Scavenger Receptor Cysteine-Rich Type 1 Protein M130
CD14 Monocytes	*EPSTI1*	Epithelial stromal interaction protein 1
	*FGL2*	Fibrinogen like 2
	*FPR1*	Formyl peptide receptor 1
	*IGSF6*	Immunoglobulin superfamily member 6
	*PHF21A*	PHD finger protein 21A
Inactivated Microglia	*IRF7*	Interferon regulatory factor 7
	*OAS1*	2’-5’-oligoadenylate synthetase 1
	*RSAD2*	Radical S-adenosyl methionine domain containing 2
Activated Microglia	*FCER1G*	Fc fragment of IgE receptor Ig
	*MSR1*	Macrophage scavenger receptor 1
	*SLAMF8*	SLAM family member 8
	*CYBB*	Cytochrome b-245, beta polypeptide
Housekeeping Genes	*ACTB*	Actin cytoplasmic 1
	*GAPDH*	Glyceraldehyde-3-phosphate dehydrogenase
	*RPLP0*	60S acidic ribosomal protein P0

## Supplementary Materials

Supplementary Materials can be found at https://www.mdpi.com/1422-0067/21/21/7924/s1.

## Abbreviations

ABC-HRP	avidin-biotin complex-horse radish peroxidase
ACTB	actin cytoplasmic 1
AKT3	V-akt murine thymoma viral oncogene homolog 3
BBB	blood brain-barrier
BST2	CD317, tethrin
CACNA1E	calcium voltage-gated channel subunit alpha 1E
CALM	calmodulin
CAMK2A	calcium/calmodulin-dependent protein kinase II alpha
CAMK4	calcium/calmodulin-dependent protein kinase IV
cAMP	cyclic adenosine monophosphateP
CCL2	C-C motif chemokine ligand 2
CCL3	C-C motif chemokine ligand 3
CCL4	C-C motif chemokine ligand 4
CCR5	C-C motif chemokine receptor 5 (gene/pseudogene)
CD163	CD163 molecule, Scavenger Receptor Cysteine-Rich Type 1 Protein M130
CD274	cluster of differentiation 274, programmed death-ligand 1
CD74	CD74 molecule, HLA class II histocompatibility antigen gamma chain
CD80	co-stimulatory molecule B7-2
CD86	cluster of differentiation 86, B7-2
CD8A	CD8a molecule, T-cell surface glycoprotein CD8 alpha chain
cDNA	complementary DNA
CFAS	Cognitive Function and Ageing Study
CNS	central nervous system
COL6A3	collagen type VI alpha 3 chain
CX3CR1	C-X3-C motif chemokine receptor 1
CYBB	cytochrome b-245, beta polypeptide
DAVID	Database for Annotation, Visualization and Integrated Discovery
DEPC	diethylpyrocarbonate
DSCL	deep subcortical lesions
ENTPD1	ectonucleoside triphosphate diphosphohydrolase 1
EPSTI1	epithelial stromal interaction protein 1
FCER1G	Fc fragment of IgE receptor Ig
FCGR1A	Fc fragment of immunoglobulin gamma Fc receptor 1A
FCGR2A	Fc fragment of immunoglobulin gamma Fc receptor 2A
FGL2	fibrinogen like 2
FPR1	formyl peptide receptor 1
GABA	gamma-aminobutyric acid
GAPDH	glyceraldehyde-3-phosphate dehydrogenase
GBP5	guanylate-binding protein 5
GEO	gene expression omnibus
GRIA	glutamate receptor, ionotropic, AMPA
GRIN1	glutamate receptor, ionotropic, N-methyl D-aspartate 1
H&E	haematoxylin and eosin
HAVCR2	hepatitis A virus cellular receptor 2
HLA-DMB	major histocompatibility complex, class II, DM beta
HLA-DPA1	major histocompatibility complex, class II, DP alpha 1
HLA-DPB1	major histocompatibility complex, class II, DP beta 1
HLA-DQB1	major histocompatibility complex, class II, DQ beta 1
HLA-DRA	major histocompatibility complex, class II, DR alpha
IFI35	interferon induced protein 35
IFI44	interferon induced protein 44
IFI44L	interferon induced protein 44-like
IFIT1	interferon induced protein with tetratricopeptide repeats 1
IFIT3	interferon induced protein with tetratricopeptide repeats 3
IFNAR1	interferon alpha receptor subunit 1
IGSF6	immunoglobulin superfamily member 6
IHC	immunohistochemistry
IKBKB	inhibitor of nuclear factor kappa B kinase subunit beta
IL1A	interleukin 1 alpha
IL1B	interleukin 1 beta
IRF7	interferon regulatory factor 7
IRF9	interferon regulatory factor 9
ISG15	interferon-stimulated gene 15
ITGAX	integrin, alpha X (complement component 3 receptor 4 subunit), CD11c
IVT	in vitro transcription
JUN	jun proto-oncogene, AP-1 transcription factor subunit
KEGG	Kyoto Encyclopedia of Genes and Genomes
LCM	laser capture microdissection
LCP2	lymphocyte cytosolic protein 2
LFB	luxol fast blue
LY6E	lymphocyte antigen 6E
MAPK	mitogen-activated protein kinase
MHC-II	major histocompatibility complex class II
MRC1	mannose receptor C-type 1
MRI	magnetic resnance imaging
mRNA	messenger ribonucleic acid
MS4A6A	membrane spanning 4-domains A6A
MSR1	macrophage scavenger receptor 1
MX1	MX dynamin like GTPase 1
OAS1	2’-5’-oligoadenylate synthetase 1
OAS2	2’-5’-oligoadenylate synthetase 2
OAS3	2’-5’-oligoadenylate synthetase 3
PCA	principal component analysis
PHF21A	PHD finger protein 21A
PI3K-Akt	phosphatidylinositol 3-kinase-protein kinase
PTGS2	prostaglandin-endoperoxide synthase 2
PVL	periventricular lesions
QC	quality control
REC	research ethics committee
RPLP0	60S acidic ribosomal protein P0
RSAD2	radical S-adenosyl methionine domain containing 2
RT	room temperature
SAMSN1	SAM domain, SH3 domain and nuclear localisation signals 1
SLAMF8	SLAM family member 8
TAC	transcriptome analysis console
TFEC	transcription factor EC
TREM2	triggering receptor expressed on myeloid cells 2
UBE2L6	ubiquitin conjugating enzyme E2 L6
USP18	ubiquitin specific peptidase 18
WM	white matter
WML	white matter lesions
XAF1	XIAP-associated factor 1
