# Supplementary material for: Transcriptomic Analysis of Age-Associated Periventricular Lesions Reveals Dysregulation of the Immune Response
Source: Int J Mol Sci. 2020 Oct 25;21(21):7924. doi: 10.3390/ijms21217924 (PMC7663268; doi:10.3390/ijms21217924)
Supplement: Supplementary file 1 [file ijms-21-07924-s001.pdf]

Supplementary Table 1. Dysregulated pathways in PVL versus non-lesional control white matter identified by KEGG pathway analysis in DAVID

Pathway	Count	p-value
hsa05215:Prostate cancer	23	1.81 $\times 10^{-6}$
hsa04510:Focal adhesion	38	4.74 $\times 10^{-6}$
hsa04612:Antigen processing and presentation	20	9.17 $\times 10^{-6}$
hsa05416:Viral myocarditis	15	1.69 $\times 10^{-4}$
hsa05332:Graft-versus-host disease	11	2.29 $\times 10^{-4}$
hsa05150:Staphylococcus aureus infection	14	3.56 $\times 10^{-4}$
hsa04940:Type I diabetes mellitus	12	4.63 $\times 10^{-4}$
hsa05200:Pathways in cancer	51	0.0013
hsa05152:Tuberculosis	28	0.0014
hsa05145:Toxoplasmosis	20	0.0016
hsa05140:Leishmaniasis	15	0.0018
hsa05212:Pancreatic cancer	14	0.0022
hsa04151:PI3K-Akt signaling pathway	45	0.0024
hsa05330:Allograft rejection	10	0.0026
hsa04145:Phagosome	24	0.0028
hsa05213:Endometrial cancer	12	0.0031
hsa05166:HTLV-I infection	35	0.0034
hsa05222:Small cell lung cancer	16	0.0039
hsa04672:Intestinal immune network for IgA production	11	0.0045
hsa05168:Herpes simplex infection	27	0.0045
hsa04520:Adherens junction	14	0.0051
hsa05169:Epstein-Barr virus infection	20	0.0053
hsa04380:Osteoclast differentiation	21	0.0054
hsa04974:Protein digestion and absorption	16	0.0054
hsa05321:Inflammatory bowel disease (IBD)	13	0.0057
hsa04152:AMPK signaling pathway	20	0.0058
hsa04514:Cell adhesion molecules (CAMs)	22	0.0064
hsa04068:FoxO signaling pathway	21	0.007
hsa04015:Rap1 signaling pathway	29	0.008
hsa05310:Asthma	8	0.01
hsa04611:Platelet activation	20	0.01
hsa04512:ECM-receptor interaction	15	0.0118
hsa05210:Colorectal cancer	12	0.0121
hsa05323:Rheumatoid arthritis	15	0.013
hsa05223:Non-small cell lung cancer	11	0.0156
hsa04915:Estrogen signaling pathway	16	0.0159
hsa04913:Ovarian steroidogenesis	10	0.0179
hsa05231:Choline metabolism in cancer	16	0.0188
hsa05211:Renal cell carcinoma	12	0.0189
hsa05142:Chagas disease (American trypanosomiasis)	16	0.024

hsa04662:B cell receptor signaling pathway	12	0.0256
hsa05320:Autoimmune thyroid disease	10	0.0257
hsa05160:Hepatitis C	19	0.0259
hsa04064:NF-kappa B signaling pathway	14	0.0264
hsa04012:ErbB signaling pathway	14	0.0264
hsa05322:Systemic lupus erythematosus	19	0.0277
hsa05146:Amoebiasis	16	0.028
hsa05164:Influenza A	23	0.03
hsa04014:Ras signaling pathway	28	0.0337
hsa04923:Regulation of lipolysis in adipocytes	10	0.0395
hsa04010:MAPK signaling pathway	30	0.0455
hsa00910:Nitrogen metabolism	5	0.0468
hsa04150:mTOR signaling pathway	10	0.048

Supplementary Table 2. Dysregulated pathways in pre-lesional versus non-lesional control white matter identified by KEGG pathway analysis in DAVID

Pathway	Count	p-value
hsa04723:Retrograde endocannabinoid signaling	32	1.39×10^{-10}
hsa04020:Calcium signaling pathway	42	3.38×10^{-9}
hsa04728:Dopaminergic synapse	34	5.29×10^{-9}
hsa04713:Circadian entrainment	28	1.41×10^{-8}
hsa04921:Oxytocin signaling pathway	36	2.98×10^{-8}
hsa04724:Glutamatergic synapse	30	6.28×10^{-8}
hsa05033:Nicotine addiction	17	6.74×10^{-8}
hsa04725:Cholinergic synapse	29	1.29×10^{-7}
hsa05031:Amphetamine addiction	21	3.45×10^{-7}
hsa05150:Staphylococcus aureus infection	18	1.45×10^{-6}
hsa04720:Long-term potentiation	20	1.63×10^{-6}
hsa05032:Morphine addiction	24	1.66×10^{-6}
hsa04727:GABAergic synapse	23	1.82×10^{-6}
hsa05140:Leishmaniasis	20	5.40×10^{-6}
hsa05169:Epstein-Barr virus infection	27	1.08×10^{-5}
hsa05014:Amyotrophic lateral sclerosis (ALS)	16	1.17×10^{-5}
hsa04612:Antigen processing and presentation	20	1.58×10^{-5}
hsa04940:Type I diabetes mellitus	14	3.11×10^{-5}
hsa05332:Graft-versus-host disease	12	5.92×10^{-5}
hsa04261:Adrenergic signaling in cardiomyocytes	27	1.02×10^{-4}
hsa04024:cAMP signaling pathway	34	1.54×10^{-4}
hsa04010:MAPK signaling pathway	40	2.23×10^{-4}
hsa05152:Tuberculosis	31	2.26×10^{-4}
hsa04726:Serotonergic synapse	22	4.37×10^{-4}
hsa04730:Long-term depression	15	4.46×10^{-4}
hsa05166:HTLV-I infection	39	4.92×10^{-4}
hsa04915:Estrogen signaling pathway	20	6.73×10^{-4}
hsa05310:Asthma	10	6.78×10^{-4}
hsa05030:Cocaine addiction	13	7.10×10^{-4}
hsa04911:Insulin secretion	18	7.82×10^{-4}
hsa05330:Allograft rejection	11	8.58×10^{-4}
hsa05416:Viral myocarditis	14	8.92×10^{-4}
hsa05168:Herpes simplex infection	30	8.94×10^{-4}
hsa05145:Toxoplasmosis	21	0.001
hsa04971:Gastric acid secretion	16	0.0012
hsa04380:Osteoclast differentiation	23	0.0017
hsa04014:Ras signaling pathway	34	0.0017
hsa04672:Intestinal immune network for IgA production	12	0.0017
hsa04022:cGMP-PKG signaling pathway	26	0.002
hsa05162:Measles	23	0.0021

hsa04145:Phagosome	25	0.0021
hsa04514:Cell adhesion molecules (CAMs)	24	0.0022
hsa04970:Salivary secretion	17	0.0024
hsa04924:Renin secretion	14	0.0027
hsa04925:Aldosterone synthesis and secretion	16	0.0035
hsa05164:Influenza A	27	0.0037
hsa04922:Glucagon signaling pathway	18	0.0043
hsa04062:Chemokine signaling pathway	28	0.0048
hsa05414:Dilated cardiomyopathy	16	0.0049
hsa04151:PI3K-Akt signaling pathway	45	0.005
hsa04360:Axon guidance	21	0.0058
hsa04721:Synaptic vesicle cycle	13	0.0068
hsa04012:ErbB signaling pathway	16	0.0069
hsa04310:Wnt signaling pathway	22	0.0071
hsa04015:Rap1 signaling pathway	30	0.0072
hsa04540:Gap junction	16	0.0077
hsa04080:Neuroactive ligand-receptor interaction	37	0.008
hsa05214:Glioma	13	0.0087
hsa04750:Inflammatory mediator regulation of TRP channels	17	0.0091
hsa04919:Thyroid hormone signaling pathway	19	0.0091
hsa04660:T cell receptor signaling pathway	17	0.011
hsa05412:Arrhythmogenic right ventricular cardiomyopathy (ARVC)	13	0.0111
hsa05320:Autoimmune thyroid disease	11	0.0121
hsa05205:Proteoglycans in cancer	28	0.0124
hsa04662:B cell receptor signaling pathway	13	0.0139
hsa05410:Hypertrophic cardiomyopathy (HCM)	14	0.0151
hsa04611:Platelet activation	20	0.0153
hsa05323:Rheumatoid arthritis	15	0.0176
hsa05146:Amoebiasis	17	0.0187
hsa04916:Melanogenesis	16	0.0235
hsa04260:Cardiac muscle contraction	13	0.0257
hsa05200:Pathways in cancer	46	0.0285
hsa04914:Progesterone-mediated oocyte maturation	14	0.0345
hsa04068:FoxO signaling pathway	19	0.0383
hsa04144:Endocytosis	30	0.0402
hsa05321:Inflammatory bowel disease (IBD)	11	0.0458
hsa05203:Viral carcinogenesis	26	0.0473
hsa04923:Regulation of lipolysis in adipocytes	10	0.0484
hsa04621:NOD-like receptor signaling pathway	10	0.0484

Supplementary Table 3. Dysregulated pathways in pre-lesional versus PVL identified by KEGG pathway analysis in DAVID

Pathway	Count	p-value
hsa04020:Calcium signaling pathway	46	4.71×10^{-11}
hsa04723:Retrograde endocannabinoid signaling	27	3.86×10^{-7}
hsa04724:Glutamatergic synapse	29	3.97×10^{-7}
hsa04713:Circadian entrainment	26	4.11×10^{-7}
hsa04921:Oxytocin signaling pathway	34	5.84×10^{-7}
hsa05033:Nicotine addiction	16	6.56×10^{-7}
hsa05032:Morphine addiction	23	9.23×10^{-6}
hsa04727:GABAergic synapse	21	3.58×10^{-5}
hsa05031:Amphetamine addiction	18	4.00×10^{-5}
hsa05014:Amyotrophic lateral sclerosis (ALS)	15	7.12×10^{-5}
hsa04720:Long-term potentiation	17	1.47×10^{-4}
hsa05412:Arrhythmogenic right ventricular cardiomyopathy	17	1.78×10^{-4}
hsa04725:Cholinergic synapse	23	2.30×10^{-4}
hsa05414:Dilated cardiomyopathy	19	3.09×10^{-4}
hsa04080:Neuroactive ligand-receptor interaction	43	3.22×10^{-4}
hsa04310:Wnt signaling pathway	26	3.83×10^{-4}
hsa04970:Salivary secretion	19	4.19×10^{-4}
hsa04024:cAMP signaling pathway	33	5.36×10^{-4}
hsa04022:cGMP-PKG signaling pathway	28	5.96×10^{-4}
hsa04390:Hippo signaling pathway	26	0.0015
hsa04261:Adrenergic signaling in cardiomyocytes	24	0.0021
hsa05410:Hypertrophic cardiomyopathy (HCM)	16	0.003
hsa00230:Purine metabolism	28	0.0031
hsa04360:Axon guidance	22	0.0035
hsa04728:Dopaminergic synapse	22	0.0039
hsa04974:Protein digestion and absorption	17	0.004
hsa04971:Gastric acid secretion	15	0.0042
hsa04911:Insulin secretion	16	0.007
hsa04978:Mineral absorption	10	0.0132
hsa04010:MAPK signaling pathway	34	0.0143
hsa04972:Pancreatic secretion	16	0.0157
hsa04512:ECM-receptor interaction	15	0.02
hsa05010:Alzheimer's disease	24	0.0224
hsa04925:Aldosterone synthesis and secretion	14	0.0244
hsa05030:Cocaine addiction	10	0.0259
hsa04114:Oocyte meiosis	17	0.034
hsa04730:Long-term depression	11	0.036
hsa04510:Focal adhesion	27	0.04
